# Neurofeedback Training of Alpha Relative Power Improves the Performance of Motor Imagery Brain-Computer Interface

**DOI:** 10.3389/fnhum.2022.831995

**Published:** 2022-04-08

**Authors:** Qing Zhou, Ruidong Cheng, Lin Yao, Xiangming Ye, Kedi Xu

**Affiliations:** ^1^Qiushi Academy for Advanced Studies (QAAS), Zhejiang University, Hangzhou, China; ^2^Zhejiang Lab, Hangzhou, China; ^3^Zhejiang Provincial Key Laboratory of Cardio-Cerebral Vascular Detection Technology and Medicinal Effectiveness Appraisal, Key Laboratory of Biomedical Engineering of Education Ministry, Zhejiang University, Hangzhou, China; ^4^Center for Rehabilitation Medicine, Rehabilitation and Sports Medicine Research Institute of Zhejiang Province, Department of Rehabilitation Medicine, Zhejiang Provincial People’s Hospital (Affiliated People’s Hospital, Hangzhou Medical College), Hangzhou, China; ^5^MOE Frontiers Science Center for Brain and Brain-Machine Integration, Zhejiang University, Hangzhou, China; ^6^Department of Neurobiology, Affiliated Mental Health Center and Hangzhou Seventh People’s Hospital, Zhejiang University School of Medicine, Hangzhou, China; ^7^The College of Computer Science, Zhejiang University, Hangzhou, China

**Keywords:** alpha relative power, motor imagery, performance variation, electroencephalogram (EEG), brain-computer interface (BCI), neurofeedback training (NFT)

## Abstract

Significant variation in performance in motor imagery (MI) tasks impedes their wide adoption for brain-computer interface (BCI) applications. Previous researchers have found that resting-state alpha-band power is positively correlated with MI-BCI performance. In this study, we designed a neurofeedback training (NFT) protocol based on the up-regulation of the alpha band relative power (RP) to investigate its effect on MI-BCI performance. The principal finding of this study is that alpha NFT could successfully help subjects increase alpha-rhythm power and improve their MI-BCI performance. An individual difference was also found in this study in that subjects who increased alpha power more had a better performance improvement. Additionally, the functional connectivity (FC) of the frontal-parietal (FP) network was found to be enhanced after alpha NFT. However, the enhancement failed to reach a significant level after multiple comparisons correction. These findings contribute to a better understanding of the neurophysiological mechanism of cognitive control through alpha regulation.

## Introduction

Motor imagery (MI) refers to the mental representation of action without engaging in its actual execution (Moran et al., 2012). In recent decades, MI has contributed to motor performance and rehabilitation, as well as to a better understanding of cognition, action, and perception (Munzert et al., 2009; MacIntyre et al., 2018). MI is also a common task in brain-computer interface (BCI) research for realizing the voluntary control of external devices (Müller-Putz et al., 2016). MI-BCI performance refers to the ability of subjects to control an MI-BCI system and is generally assessed through decoding accuracies. MI-BCI performance shows a significant intra- and inter-subject variability (Ahn and Jun, 2015; Saha and Baumert, 2020; Zhou et al., 2021), which impedes its wide adoption for BCI applications. Understanding the neural mechanisms behind such variability is a promising approach that will aid in solving the problem (Xu et al., 2021).

Previous studies are inclined to the view that a positive association exists between alpha rhythm and MI-BCI performance. An earlier study of 80 subjects used the sensorimotor rhythm (SMR, including the alpha and beta bands) from a 2 min resting state to predict MI-BCI performance (Blankertz et al., 2010). Another intra-subject study reported that trials with higher SMR power in the 1 s interval preceding the cue yielded better performance (Maeder et al., 2012). Further, some researchers found that lower resting-state alpha-band relative power (RP) may indicate poor MI performers (Ahn et al., 2013; Kwon et al., 2020). Our previous study suggests that the positive correlation between alpha-band RP and MI-BCI performance was consistent in inter-subject and inter-session analysis, especially over the frontal lobes (Zhou et al., 2021). These results indicate that the strength of alpha rhythm in the resting state might reflect an unstable cognitive state, which results in the variation of MI-BCI performance.

Although understanding how alpha rhythm controls the cognitive state remains elusive, a positive relationship between higher resting-state alpha activity and a better cognitive level has been reported in many studies. An experiment on cognitive task performance found that resting-state alpha power was positively correlated with attention-span scores over 82 healthy adults (Mahjoory et al., 2019). Other studies have reported that people with amnestic mild cognitive impairment (aMCI) or subjective cognitive decline (SCD) had a lower resting-state alpha band RP (Bian et al., 2014; López-Sanz et al., 2016). Some researchers have suggested that alpha power sustains attention by regularly purging task-irrelevant information and neural noise (Sadaghiani and Kleinschmidt, 2016), which may explain the relevance of a higher resting-state alpha power to better MI or cognitive tasks.

Neurofeedback is an operant conditioning technique that could help subjects self-regulate targeted brain activity patterns (Wood et al., 2014; Sitaram et al., 2017). Today, neurofeedback training (NFT) has been widely investigated for its possible applications in cognitive enhancement and psychiatric amelioration (Vernon, 2005; Gruzelier, 2014; Micoulaud-Franchi et al., 2015). Numerous studies have demonstrated that alpha NFT can significantly and independently improve resting-state alpha band power (Wan et al., 2014; Ossadtchi et al., 2017). In addition, alpha NFT has been proven to enhance attentional control (Berger and Davelaar, 2018) and has a positive effect on working memory (WM) or episodic memory (EM) (Hsueh et al., 2016; Yeh et al., 2021). These results may support the view that the up-regulation of alpha oscillations contributes to efficient neurocognitive processing (Berger and Davelaar, 2018).

Some studies have reported that alpha NFT can enhance cognitive performance assessed by a mental rotation test (MRT) (Hanslmayr et al., 2005; Zoefel et al., 2011) or matrix rotation task (Reis et al., 2016). MRT scores were found to be strongly correlated with MI-BCI performance (Jeunet et al., 2015). Researchers explain that the MRT score reflects the user’s spatial ability, which is one of the potential MI-BCI performance predictors (Jeunet et al., 2016). In addition, some researchers found that alpha NFT can be used to enhance the event-related desynchronization (ERD) of a motor execution task in healthy subjects (López-Larraz et al., 2012) and motor attempts in spinal cord injury patients (López-Larraz et al., 2019). ERD is a common neurological manifestation in both motor imagery and motor execution, and it is also an important feature of MI-BCI performance decoding. These findings suggest that alpha NFT may be a promising way to improve MI-BCI performance.

A previous study showed the feasibility of improving MI-BCI performance through alpha NFT (Bamdadian et al., 2015). In their study, subjects achieved better MI-BCI performance and higher resting-state alpha RP compared to the control group after a 4-week NFT. However, a limitation of this study was its relatively small sample sizes and inadequate analysis. Besides, the alpha-band indicators used in training and evaluation were inconsistent, the power of extracted alpha spatial-spectral decomposition (SSD) components was used for training whereas alpha RP was used for evaluation. Further, neural feedback inefficacy was a significant problem in the previous study. In the experimental group, 2 out of 6 subjects showed almost no improvement in MI-BCI performance. A review in the previous paper has discussed this inefficacy problem of NFT, which is much more widespread than currently realized and warrants further exploration (Alkoby et al., 2018).

Alpha-band RP has been proved to be a promising stable neurophysiological indicator for MI-BCI performance in our previous work (Zhou et al., 2021). However, few studies have used alpha-band RP as the target signal of NFT. Therefore, the purpose of this study is to develop a novel NFT system based on alpha-band RP and explore: (1) the effectiveness of enhancing resting-state alpha power and improving MI-BCI performance through alpha NFT, (2) individual differences and the problem of NFT inefficacy, and (3) the physiological mechanism of the impact of alpha rhythm on cognitive control.

## Materials and Methods

### Subjects

Eighteen healthy subjects (8 males, mean age: 23.46 ± 1.51 years, range: 21–28 years, all right-handed) participated in our study. None of the participants reported a history of psychiatric or neurological disorders. One of them had participated in MI studies before. The study was designed and conducted according to the *Declaration of Helsinki* and approved by the Human Research Ethics Committee of the Second Affiliated Hospital of Zhejiang University School of Medicine. All subjects were asked to read and sign an informed consent form before the experiment and received financial compensation after the experiments for their time and effort.

### Experimental Protocol

**FIGURE 1:**
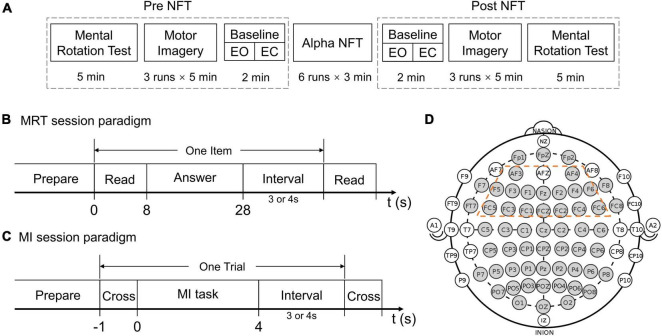
**(A)** The experimental procedure. **(B)** Mental rotation test (MRT) session paradigm. **(C)** Motor imagery (MI) session paradigm. **(D)** The electrodes (gray) which were recorded in the international 10–20 system – the 16 electrodes circled by a dotted orange line were chosen as the target training area from the frontal lobe.

During the experiment, subjects sat in a comfortable chair in front of a computer screen and were instructed to relax their arms and minimize any physical movement or eye blinking throughout the electroencephalogram (EEG) recording process. Each subject participated in a seven-session experiment in 1 day. The participants could take a short rest between sessions. The total experimental time of each subject was less than 1.5 h, excluding the preparation time before the experiment. Figure 1A illustrates the procedure of the experimental paradigm and the separate time of each session. The seven-session experiment consisted of two MRT sessions, two MI sessions, two baseline sessions, and an alpha NFT session.

Each of the two MRT sessions comprised ten items, and one item comprised four options, two of which were correct. One point was counted only when both correct options were selected. Subjects were asked to read and answer each item in 8 and 20 s, respectively (Figure 1B). After the “answer” cue, subjects could submit their answers by pressing corresponding buttons on a keyboard. No point was counted if no submission was made when the timeout occurred. In this study, a modified-version MRT composed of two equally difficult parts was used (Moè, 2021), respectively, assessing mental rotation abilities before and after the alpha NFT.

For the two MI sessions, each session was organized into 3 runs, each run lasting 5 min. During each run, subjects were asked to perform left-hand MI, right-hand MI, or idle tasks according to the on-screen cue. At the beginning of each trial, a white fixation cross appeared in the middle of the black screen and stayed for 1 s. During the left/right-hand MI task, a 3D simulation graph of the left/right hand was shown on the corresponding side of the cross, and subjects were instructed to perform kinaesthetic MI. During the idle task, the white cross turned green, and subjects were instructed to relax and to not think about anything. Each trial lasted 8 or 9 s (Figure 1C). There were 12 trials per task, a total of 36 trials in each run, and 108 trials in each session. The tasks were presented in random order. No feedback was provided to the subjects during the MI sessions.

Two-minute baselines were recorded immediately before and after the alpha NFT. Each baseline session was organized into a 1 min, eye-open (EO), resting-state run and a 1 min, eye-closed (EC), resting-state run.

The alpha NFT session was organized into 6 runs, each run lasting 3 min. During each run, subjects received real-time feedback of alpha changes – provided visually as a vertical bar on the screen. The ordinate value of the bar was proportional to the deviation of the alpha indicator from the baseline. The bar color turned green or red when the alpha indicator was higher or lower than the baseline, respectively. Subjects were instructed to remain relaxed to increase their alpha indicator during the three continuous minutes. Additionally, subjects were given smileys as emotional support when they performed well within a short time (details in section “Alpha Neurofeedback Training System Design”).

### Electroencephalogram Recording and Pre-processing

Electroencephalogram recording was performed using a 64-channel Synamps2 system (Neuroscan, Inc., Victoria, Australia) with a sampling frequency of 500 Hz. 60 EEG scalp electrodes were prior-selected according to the international 10–20 system, 4 electrodes were then excluded due to the poor signal quality. The remaining 56 channels were marked in gray as shown in Figure 1D. The reference electrode was placed on the top of the head between Cz and CPz, and the ground electrode AFz was placed on the medial frontal area of the head. A horizontal electrooculogram (EOG) and vertical EOG were recorded using the same system. A band-pass filter between 0.5 and 100 Hz and a notch filter of 50 Hz were applied directly to the amplifier.

### Alpha Neurofeedback Training System Design

As mentioned above, to help subjects upregulate alpha band power, the alpha indicator was calculated and fed back in real-time during the alpha NFT session. In this study, the deviation of alpha relative power (ARP) from the baseline was used as the alpha indicator. Before the alpha NFT session, the individual alpha frequency (IAF) from the eye-closed (EC) baseline was calculated using an automated and open-source method (Corcoran et al., 2018). After this, the ARP of the eye-open (EO) baseline before NFT was calculated as ARP_baseline_. During the alpha NFT session, the continuous EEG recording was segmented into 1 s epochs and updated every 100 ms. The ARP of the EEG epoch was calculated as ARP_epoch_. The ARP and the deviation of ARP (ΔARP) from the baseline were calculated as follows:


(1) $$\begin{array}{c} \text{ARP}=\frac{{\text{P}}_{\text{alpha}}}{{\text{P}}_{\text{total}}} \end{array}$$

(2) $$\begin{array}{c} \Delta \,\text{ARP}=\frac{{\text{ARP}}_{\text{epoch}}-{\text{ARP}}_{\text{baseline}}}{{\text{ARP}}_{\text{baseline}}}. \end{array}$$

P_alpha_ and P_total_ indicate the absolute power of the alpha band from (IAF-2, IAF+2) Hz and the total band from (1, 50) Hz, respectively. The multitaper method was used to calculate the power spectral density (PSD) (Percival and Walden, 1993), and the integration method was used to obtain the target band power. The multitaper function was implemented through the *MNE-Python* package (Gramfort et al., 2013). In this study, 16 frontal lobe electrodes were selected to obtain the averaged ARP used in the alpha NFT session (Figure 1D).

**FIGURE 2:**
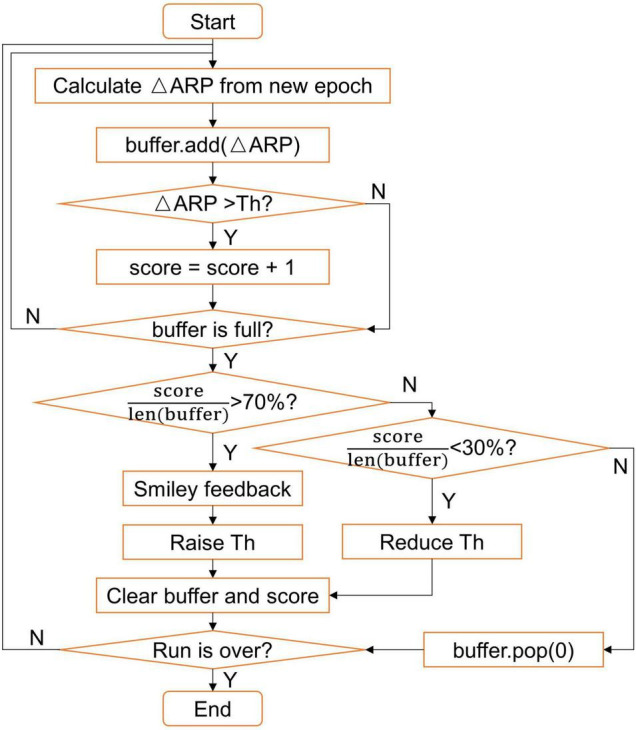
Flow chart of the dynamic threshold strategy.

The ΔARP value was mapped to the ordinate value of the visual feedback bar in real-time, and the sign of the ΔARP (positive or negative) corresponded to the bar color (green or red). If the ΔARP value of most epochs within a few seconds exceeded the custom-set threshold, a smiley would be presented on the screen, indicating good performance. A dynamic threshold strategy was designed to better adapt to the subjects during each run (see Figure 2). In this study, the maximum length of the buffer was set to 100 (about 10 s), the initial threshold (Th) was set to 10%, the stride of raising or reducing threshold was set to 1%, and the threshold was set to no less than 1%. The feedback would be paused briefly if EOG artifacts were detected.

### Feature Extraction and Classification

The result of offline classification using the whole brain electrodes was used to measure MI-BCI performance. First, the EEG signal of the MI session was band-pass filtered by a linear phase, finite impulse response (FIR) filter between 8 and 30 Hz. Then, the signals were extracted from 0 to 4 s after task onset. The three-class classification (i.e., left hand, right hand, and idle task) and three binary classifications were considered. EEG epochs were spatially filtered using the one versus rest (OVR) Common Spatial Pattern (CSP) algorithm (Ang et al., 2012). After that, the features were fed into the Support Vector Machine (SVM) algorithm with a radial basis function kernel to generate subject-specific models. A 10-fold cross-validation (CV) procedure was performed to validate the results.

### Power Spectral Analysis

The power and relative power (RP) of different bands were used to investigate changes of brain rhythms. The individual frequency bands included the delta band from (1, IAF-6) Hz, theta band from (IAF-6, IAF-2) Hz, alpha band from (IAF-2, IAF+2) Hz, beta band from (IAF+2, 30) Hz, and the low gamma band from (30, 50) Hz (Escolano et al., 2014a). The calculation of RP was similar to ARP from the online, alpha NFT session, the ΔRP was calculated as follows:


(3) $$\begin{array}{c} \Delta \,\text{RP}=\frac{\text{RP}-{\text{RP}}_{\text{baseline}}}{{\text{RP}}_{\text{baseline}}}. \end{array}$$

For the baseline session, we investigated the ΔRP of five bands. The RP and RP_baseline_ indicated the specific band RP from post- and pre-NFT baseline sessions, respectively.

For the alpha NFT session, only the alpha band ΔRP of six runs was analyzed separately. The RP and RP_baseline_ indicated the individual alpha band RP from the specific run and the previous baseline session before NFT.

For the MI session, the EEG signal from −3 to 4 s was extracted. Each trial was split into a pre-task stage (−3 – 0 s) and a task stage (0 – 4 s), and further grouped by the type of task. The power was averaged over three runs of each session. The ERD of each task was calculated as follows:


(4) $$\begin{array}{c} \mathrm{ERD}=\frac{{\text{P}}_{\text{task}}-{\text{P}}_{\text{pretask}}}{{\text{P}}_{\text{pretask}}}, \end{array}$$

where P_task_ and P_pretask_ represent the absolute power of the individual alpha band from the task and pre-task stage, respectively.

### Functional Connectivity Analysis

Resting-state functional connectivity (FC) was analyzed to investigate changes in the brain networks. To map the connectivity between the bilateral brain regions, the eight central electrodes (i.e. Fpz, Fz, FCz, Cz, CPz, Pz, POz, and Oz) were excluded. 24 electrodes of each side with a total of 48 electrodes were selected from EO baseline session. The functional connectivity matrix was calculated by the phase lag index (PLI) and Imaginary Coherence (imCoh), which are both very commonly used measurements to date. Previous studies have suggested that PLI is less affected by the influence of common sources and active reference electrodes (Stam et al., 2007), while imCoh is weakly affected by volume conduction and spatial leakage (Nolte et al., 2004). The implementation of algorithms and the drawing of brain network connection maps were carried out through the *MNE-Python* package (Gramfort et al., 2013).

### Statistical Analysis

A paired *t*-test was applied to statistically analyze the significance of differences from before and after the alpha NFT stage. Pearson correlation was performed to analyze the correlation between ΔARP and the performance changes within subjects. A false discovery rate (FDR) of α = 0.05 was used to correct the significance level for multiple comparisons. The statistical and correlational methods used in this paper were implemented using the Python package *Pingouin* (Vallat, 2018).

## Results

### Motor Imagery and Mental Rotation Test Performance Analysis

**FIGURE 3:**
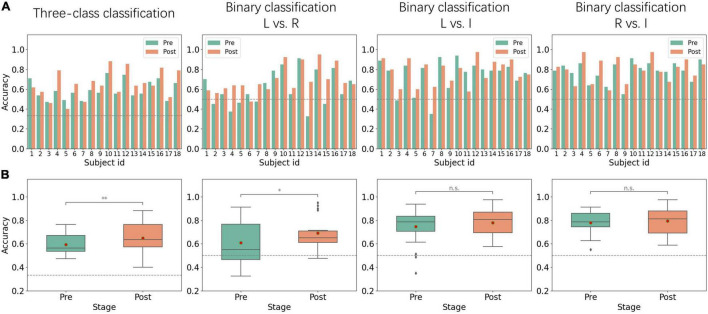
Performance from pre-NFT (Pre) and post-NFT (Post) MI sessions. **(A)** Histogram of inter-subject classification accuracy – the dotted gray line represents the random levels of 33.33 and 50%. **(B)** Boxplots of classification accuracy - the red dot represents the average value: L vs. R, left-hand MI versus right-hand MI; L vs. I, left-hand MI versus idle task; R vs. I, right-hand MI versus idle task; *p < 0.05; ^**^p < 0.01; n.s., no significance.

Figure 3 shows the classification results of MI sessions before and after alpha NFT. For the three-class classification, all of the subjects achieved performances higher than the random level (33.33%). Overall, the average classification accuracy was 59.29 ± 9.17% before alpha NFT and 64.78 ± 13.45% after alpha NFT, which shows a significant improvement of 5.48 ± 7.76% (*p* < 0.01). However, there remained 30% of the 18 subjects showed no increase in MI-BCI performance.

For binary classification, left-hand MI versus right-hand MI (L vs. R), left-hand MI versus idle task (L vs. I), and right-hand MI versus idle task (R vs. I) were considered. L vs. R classification results show that average accuracy was 60.83 ± 16.94% before alpha NFT and 69.08 ± 13.12% after alpha NFT, which shows a significant improvement of 8.25 ± 12.66% (*p* < 0.05). Whereas L vs. I and R vs. I classification results show that average accuracies were 74.51 ± 15.36% and 77.92 ± 9.78% before alpha NFT, and 77.78 ± 11.99% and 79.47 ± 11.54% after alpha NFT, the improvement failed to achieve a significant level.

For MRT results, the full score was 10, and the average score of subjects was 5.33 ± 2.13 before alpha NFT and 5.22 ± 2.15 after alpha NFT; no significant difference was found. The timeout rate was 4.17%.

### Relative Power Changes and Correlation Analysis

**FIGURE 4:**
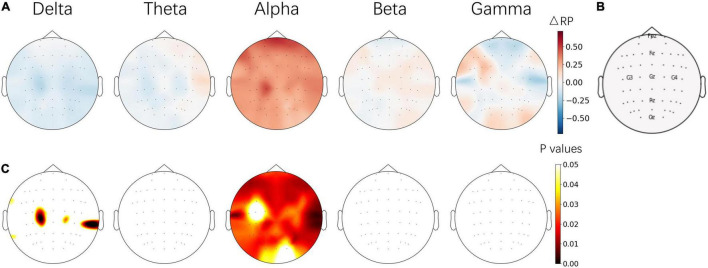
**(A)** Topographical images of ΔRP between the pre- and post-NFT baseline sessions from 5 frequency bands. **(B)** Position of electrodes. **(C)** The significant level of ΔRP from paired *t*-test – the *p* values of electrodes were corrected by FDR. The images in **(A,C)** share the same color bars on the right, respectively.

The average IAF of the subjects was stable between pre- and post-NFT EC baseline sessions, which were 10 ± 0.49 Hz and 9.72 ± 0.53 Hz, respectively. To evaluate trainability and independence, the ΔRP of five frequency bands between pre- and post-NFT EO baseline sessions were calculated. The difference of each channel was corrected by FDR. The topographical images were plotted as shown in Figure 4. The results show that alpha-band RP (ARP) was significantly increased on almost the whole brain, especially on the frontal lobe. Few significances of ΔRP were found from the delta band and no significant difference was found from other frequency bands.

**FIGURE 5:**
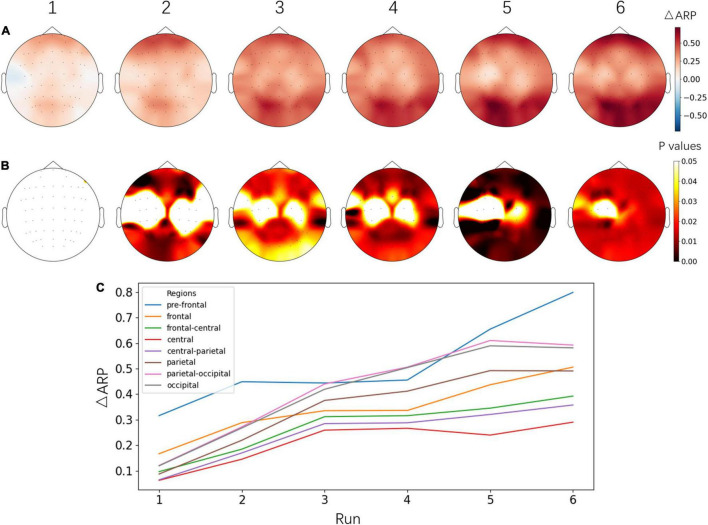
**(A)** Topographical images of ΔARP between each alpha NFT run and the baseline session before NFT. **(B)** The significant level of ΔARP from the paired *t*-test – the *p* values of electrodes were corrected by FDR. The images in **(A,B)** share the same color bars on the right, respectively. **(C)** Line plot of ΔARP from different brain regions across six alpha NFT runs. Electrodes were grouped into 8 brain regions by their name and averaged in each region. Fp and AF: pre-frontal, F: frontal, FC: frontal- central, C: central, CP: central- parietal, P: parietal, PO: parietal-occipital, O: occipital area.

To evaluate the progress of the NFT, the average ARP values from 6 runs of the alpha NFT session were computed separately, then ARP changes between each run and the previous EO baseline session were plotted in Figure 5. An obvious increasing trend was observed from run 1 to run 6. ARP was significantly increased on almost the whole brain, especially on the pre-frontal and parietal-occipital lobes.

**FIGURE 6:**
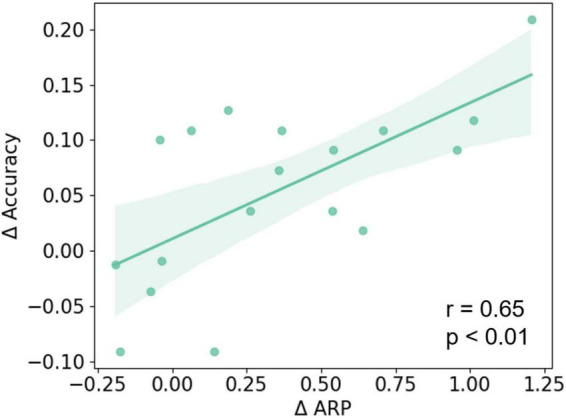
The Pearson correlation between ΔARP and changes of MI-BCI performance (ΔAccuracy). A significant positive correlation was found between the ΔARP and ΔAccuracy of the three-class classification (*r* = 0.65; *p* < 0.01).

After that, ΔARP was evaluated by the relative difference between post- and pre-NFT EO baseline sessions, and was averaged across 16 frontal lobe electrodes. Then, we applied the Pearson correlation to investigate the relationship between ΔARP and MI-BCI performance changes. The results show that ΔARP was significantly and positively correlated with MI-BCI performance changes (see Figure 6) (*r* = 0.65; *p* < 0.01). However, when considering MRT performance, no significant correlation was found between MRT score changes and ΔARP or MI-BCI performance changes.

### Event-Related Desynchronization Analysis

**FIGURE 7:**
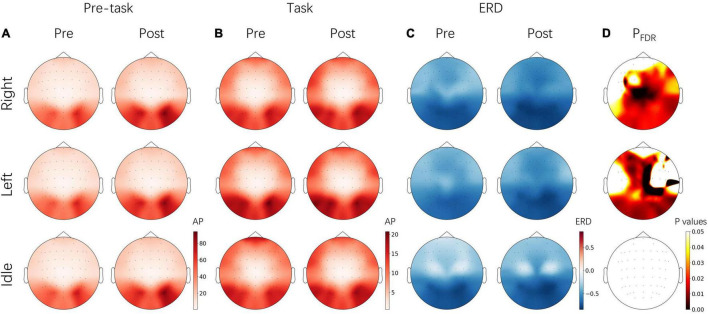
Topographical images of alpha-band absolute power (AP) and ERD from pre-NFT (Pre) and post-NFT (Post) MI session and three tasks, respectively. **(A)** The AP from the pre-task stage (–3 – 0 s). **(B)** The AP from the task stage (0 – 4 s) **(C)** The ERD. **(D)** The significant difference of ERD from the paired *t*-test – the *p* values of electrodes were corrected by FDR. The images of each panel share the same color bars on the bottom right, respectively. right, right-hand MI; left, left-hand MI; idle, idle task.

The changes of absolute band power and ERD values between pre- and post-NFT MI sessions were also analyzed from three tasks, respectively. As shown in Figure 7, after alpha NFT, pre-task stage alpha-band power was increased, especially over occipital lobes, and slightly increased over the prefrontal lobe – while no obvious changes were observed from the task stage after alpha NFT. Further, the ERD calculated was found to be enhanced from both the left-hand and right-hand MI, especially over the contralateral parietal lobe. And a higher significance level of ERD changes was found over the contralateral primary motor cortex (M1).

### Functional Connectivity Analysis

**FIGURE 8:**
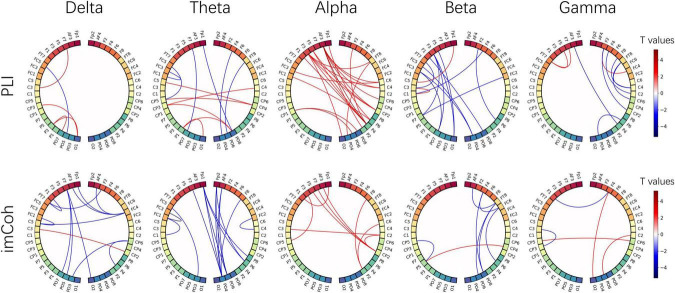
The functional connectivity difference from five frequency bands, calculated by the phase lag index (PLI) and imaginary coherence (imCoh). The nodes correspond to 48 electrodes from bilateral hemispheres, and the links represent the statistical values resulting from a paired *t*-test between pre- and post-NFT baseline sessions. The red and blue color of the line indicates that the connection was enhanced or weakened after alpha NFT, respectively. Only significant links (*p* < 0.01) are illustrated -whereas no significance was found after the FDR correlation.

The EEG signals from the EO baseline sessions were further analyzed for variation of the resting state brain network. The 1 min baseline signal was divided into 12 segments, 5 s per segment. The functional connectivity matrixes were calculated by PLI and imCoh and averaged across segments. Then the statistical *T* values were calculated between post- and pre-NFT baseline sessions. Figure 8 shows the *T* values resulting from the paired *t*-test, only significant links (*p* < 0.01) are illustrated. However, no significance was found after the FDR correlation. The red and blue color of the line indicates that the connection was enhanced or weakened after alpha NFT, respectively. Both PLI and imCoh results show more red lines than blue lines in the alpha band, indicating that alpha band connectivity enhanced after alpha NFT, especially between the left frontal lobe and the right parietal lobe. Additionally, more blue lines than red lines were observed in the delta and theta band from imCoh results, the beta band in PLI and imCoh results, and the gamma band from PLI results, which showed that the functional connection weakened in different degrees from these 4 frequency bands.

## Discussion

### Effectiveness of Alpha Neurofeedback Training

The amplitudes (absolute or relative) of the monitored EEG bands recorded on the baseline before and after the entire training were a typical evaluation of NFT efficacy (Rogala et al., 2016). In this study, the resting-state alpha rhythm of the baseline session was increased successfully after alpha NFT, and the enhancement was band-specific (Figure 4). This result confirmed the conclusion that alpha rhythm could be upregulated independently from other frequency bands (Zoefel et al., 2011). Additionally, although a significant difference was found over almost the entire brain, the ΔRP increased mostly over the frontal lobe, which was used as the target modulating region of alpha NFT in this study. In the course of the alpha NFT session, ARP shows a gradually increasing trend across six runs, mainly over the frontal and parietal-occipital lobes (Figure 5). Notably, an increase of ARP over the parietal-occipital lobe appeared in the alpha NFT sessions and disappeared in the following baseline session. This result may be explained by the fact that the alpha NFT task required more visuospatial attention, thus increasing the alpha power over the parietal-occipital lobe (Kelly et al., 2006).

Our previous study showed no obvious MI-BCI performance improvement across six runs in 1 day or even across seven sessions in multiple days without feedback (Zhou et al., 2021), suggesting that subjects found it difficult to regulate their brain rhythms to successfully reach the expected output without feedback. In this study, MI-BCI performance was significantly improved after alpha NFT, especially between left-hand and right-hand MI tasks. This result is consistent with a previous study (Bamdadian et al., 2015), in which only binary classification was performed. Remarkably, only one alpha NFT session lasting 18 min was applied in our study, whereas 12 sessions were used in the previous MI study, and multiple sessions are usually used in cognitive studies (Yeh et al., 2021). Hence, our results demonstrate the effectiveness of our novel NFT system based on alpha-band RP in improving MI-BCI performance after short-term training. In addition, a previous NFT study instructed subjects to enhance lateralized relative ERD by performing MI, thus improving MI-BCI performance (Wang et al., 2019). Different from their paradigm, the self-regulation in our NFT session is independent of the MI task. The proposed alpha NFT paradigm may be easier and more operable, and it is also potential to be combined with the previous studies in the future.

### Individual Difference of Alpha Neurofeedback Training

Previous reviews have pointed out that most studies have focused on finding group differences rather than on revealing individual differences. However, from the summary of Alkoby et al. (2018), about 16 to 57% of the subjects failed to modify their brain activity in the NFT field. Hence, they highlighted the importance of a wide reporting and investigation of individual differences from neurofeedback learning. Further, previous studies have also found that the effectiveness of brain activity modulation through NFT may influence behavioral changes. It has been reported that only subjects who successfully increased their alpha power after NFT could reach a higher learning efficiency (Brickwedde et al., 2019) or performed better on memory tasks (Hsueh et al., 2016), mental rotation tasks (Hanslmayr et al., 2005; Zoefel et al., 2011), and in clinical behaviors (Deiber et al., 2020). In this MI study, some of the subjects did not perform well at the beginning but achieved a considerable improvement after alpha NFT. In addition, the subjects with more alpha band up-regulation were found to have better MI-BCI performance improvement (Figure 6). These findings indicate that alpha NFT is a promising way to address to some extent the inefficacy problem.

The review in the previous paper has outlined the important characteristics regarding the set-up of neurofeedback protocols and has highlighted that personalized intervention should be considered in neurofeedback system design (Enriquez-Geppert et al., 2017). In this study, the individualized frequency interval was extracted for the target alpha-band feature. Further, proportional feedback rather than binary feedback was applied and combined with the corresponding side simulation hand image. Continuous feedback rather than discrete feedback was applied, and the latency of real-time feedback was about 100 ms. This is because a previous review recommended that the feedback latency should not exceed 250–350 ms (Sherlin et al., 2011). Also, a shorter delay was reported to facilitate the efficient learning of alpha regulation (Belinskaia et al., 2020). Moreover, the NFT threshold was adjusted dynamically within the runs to adapt to the personalized learning curve. Our results prove that an alpha NFT system using such advanced methods is feasible and effective.

Mental strategy is also an important factor for affecting the individual NFT effect. In the alpha NFT session of our study, subjects were instructed to remain relaxed, keep mentally focused, and physically relaxed, which have been reported as effective strategies (Kober et al., 2013). Besides, psychological variables such as motivation or mood should also be systematically manipulated in future research to improve the efficacy of NFT (Kadosh and Staunton, 2019).

### Physiological Mechanism of Alpha Neurofeedback Training

Previous studies have suggested that increased alpha power will lead to decreased neuronal activity – the inhibition is cyclic and referred to as pulsed inhibition (Jensen and Mazaheri, 2010). Top-down modulatory and cognitive control was realized through this mechanism of alpha oscillations. In this study, alpha-band power was found to have successfully increased before tasks from MI session after alpha NFT (Figure 7). This increase of pre-task baseline contributed to the significant enhancement of ERD. Similar results were reported in previous studies focused on motor execution (ME) tasks, in which larger alpha power before tasks as well as larger ERD during tasks was found in the alpha NF group (López-Larraz et al., 2012; Escolano et al., 2014b). This enhancement of alpha rhythms might reveal better cognitive control and further cause an improvement in classification performance from ME or MI tasks.

A recently proposed viewpoint suggests that brain networks use different features of alpha oscillations to modulate information processing (Klimesch et al., 2007; Sadaghiani and Kleinschmidt, 2016; Peylo et al., 2021). According to Sadaghiani’s model, the widespread enhancement of alpha oscillations could regularly purge accumulated task-irrelevant and distracting information and neural noise, thereby maintaining alertness. Another study demonstrated the hypothesis that alpha power directly relates to distractor suppression and operates independently from target selection (Wöstmann et al., 2019). Meanwhile, focal alpha desynchronization (also described as focal disinhibition) reflects the top-down guidance of selective attention, which permits the prolonged accumulation of local activity and enhances information processing in these areas, such as the ERD over contralateral motor cortex in MI tasks in our study, or evoked alpha desynchronization over contralateral occipital cortex in visuospatial attention tasks (Lobier et al., 2018). In addition, this theory suggests that frontal-parietal (FP) network activity can modulate long-range phase-locking in the alpha band, which facilitates information exchange and further enables phasic adaptive control (Sadaghiani et al., 2012). In our FC analysis, the alpha-band connection between the frontal and parietal lobe tended to be enhanced after alpha NFT (Figure 8), which may be evidence of how alpha NFT promotes adaptive cognitive control.

### Limitations and Further Research

One of the limitations of this study is the short training period. Although it achieved the expected changes in neurophysiological patterns and an overall improvement of MI-BCI performance, the binary classification result between MI and idle tasks was found to have no significant improvement. The reason may be due to the relatively higher performance before NFT and the insufficient training time. L vs. I and R vs. I classification results were 74.51 ± 15.36% and 77.92 ± 9.78% before alpha NFT, much higher than L vs. R results (60.83 ± 16.94%), which may reduce the room for improvement. In a previous study, after a 4-month mindfulness and BCI training, participants successfully increased alpha-band activity and BCI performance between MI and rest tasks (Stieger et al., 2020). Hence, the effectiveness of long-term alpha NFT for MI-BCI performance should be further investigated in future research. In addition, another limitation in this study is the lack of control groups. Appropriate control groups (active or passive) should be designed in future NFT studies (Rogala et al., 2016).

Notably, still about 30% of the subjects showed no obvious improvement of either alpha band power or MI-BCI performance. It remains unclear whether the cause of these “non-responders” was an inappropriate feedback approach or fundamental differences in neurophysiology (Thompson, 2019). The problem of NFT inefficiency should be taken into account and widely reported and investigated in future studies. Further, no obvious improvement was found in MRT performance, which is possibly due to the user-unfriendly design and the limited time of the MRT session, which may have increased the subjects’ anxiety and stress. Thus, personalized and humanized NFT procedures should be widely adopted to achieve efficient brain regulation and ultimately improve behavior performance.

## Conclusion

In summary, our findings demonstrate the effectiveness of alpha NFT for independent up-regulation of alpha rhythms and the improvement of MI-BCI performance. Additionally, individual differences in the NFT effect are reported in this paper. Our study complements earlier studies and shows that alpha NFT is a promising protocol for solving the problem of inefficacy, enhancing cognitive function, and further improving behavior performance. Moreover, the neurophysiological results confirm the theoretical mechanism of alpha oscillation involved in cognitive control. Our study can be an inspiration for future studies to systematically explore the effect of alpha NFT on various tasks and improve the understanding of cognitive control mechanisms.

## Data Availability Statement

The raw data supporting the conclusions of this article will be made available by the authors, without undue reservation.

## Ethics Statement

The studies involving human participants were reviewed and approved by Human Research Ethics Committee of the Second Affiliated Hospital of Zhejiang University School of Medicine. The patients/participants provided their written informed consent to participate in this study.

## Author Contributions

QZ and RC conducted the study. QZ performed the data analysis and wrote the text of the article. LY, XY, and KX supervised the study and proofread the article. All authors read and approved the final article.

## Conflict of Interest

The authors declare that the research was conducted in the absence of any commercial or financial relationships that could be construed as a potential conflict of interest.

## Publisher’s Note

All claims expressed in this article are solely those of the authors and do not necessarily represent those of their affiliated organizations, or those of the publisher, the editors and the reviewers. Any product that may be evaluated in this article, or claim that may be made by its manufacturer, is not guaranteed or endorsed by the publisher.
