# Corrigendum: Neurofeedback training of alpha relative power improves the performance of motor imagery brain-computer interface

**DOI:** 10.3389/fnhum.2022.977387

**Published:** 2022-07-14

**Authors:** Qing Zhou, Ruidong Cheng, Lin Yao, Xiangming Ye, Kedi Xu

**Affiliations:** ^1^Qiushi Academy for Advanced Studies (QAAS), Zhejiang University, Hangzhou, China; ^2^Zhejiang Lab, Hangzhou, China; ^3^Zhejiang Provincial Key Laboratory of Cardio-Cerebral Vascular Detection Technology and Medicinal Effectiveness Appraisal, Key Laboratory of Biomedical Engineering of Education Ministry, Zhejiang University, Hangzhou, China; ^4^Center for Rehabilitation Medicine, Rehabilitation and Sports Medicine Research Institute of Zhejiang Province, Department of Rehabilitation Medicine, Zhejiang Provincial People's Hospital (Affiliated People's Hospital, Hangzhou Medical College), Hangzhou, China; ^5^MOE Frontiers Science Center for Brain and Brain-Machine Integration, Zhejiang University, Hangzhou, China; ^6^Department of Neurobiology, Affiliated Mental Health Center and Hangzhou Seventh People's Hospital, Zhejiang University School of Medicine, Hangzhou, China; ^7^The College of Computer Science, Zhejiang University, Hangzhou, China

**Keywords:** alpha relative power, motor imagery, performance variation, electroencephalogram (EEG), brain-computer interface (BCI), neurofeedback training (NFT)

In the published article, there was an error in affiliations 1 and 2. Instead of “^1^ Zhejiang Lab, Hangzhou, China, ^2^ Qiushi Academy for Advanced Studies (QAAS), Zhejiang University, Hangzhou, China,” it should be “^1^ Qiushi Academy for Advanced Studies (QAAS), Zhejiang University, Hangzhou, China, ^2^ Zhejiang Lab, Hangzhou, China.”

The authors apologize for this error and state that this does not change the scientific conclusions of the article in any way. The original article has been updated.

## Publisher's note

All claims expressed in this article are solely those of the authors and do not necessarily represent those of their affiliated organizations, or those of the publisher, the editors and the reviewers. Any product that may be evaluated in this article, or claim that may be made by its manufacturer, is not guaranteed or endorsed by the publisher.

